# Hepatic stellate cells and parasite-induced liver fibrosis

**DOI:** 10.1186/1756-3305-3-60

**Published:** 2010-07-21

**Authors:** Barrie Anthony, Jeremy T Allen, Yuesheng S Li, Donald P McManus

**Affiliations:** 1 Queensland Institute of Medical Research, Post Office Royal Brisbane Hospital, Brisbane, Q 4029, Australia; 2 Centre for Parasitology and Disease, Biomedical Sciences Research Institute, University of Salford, Manchester, M5 4WT, UK

## Abstract

Fibrogenesis is a common feature of many diseases where there is severe insult to the liver. The hepatic stellate cell trans-differentiation into a myofibroblast has been identified as an important event in liver fibrogenesis and has been well investigated over the last few years in a number of liver diseases. The trans-differentiation process can be monitored *in vitro *by evaluation of biomarkers that are characteristic of normal quiescent hepatic stellate cells or activated myofibroblasts. Two major parasitic diseases associated with liver injury and fibrosis are schistosomiasis and echinococcosis. Recent studies have highlighted a role for activated hepatic stellate cells in both murine and human schistosomiasis as well as demonstrating that schistosome antigens are able to regulate this trans-differentiation process. Study of the hepatic stellate cell and its interaction with parasite-derived antigens may be pivotal in our understanding of the pathology associated with schistosomiasis and other parasitic diseases, including echinococcosis, as well as revealing new information on the trans-differentiation process in this cell type.

## Introduction

Fibrosis is a pathological process arising from abnormal and continuous wound repair processes and is a well recognised feature of several important parasitic infections. Hydatid cysts, arising from infection with metacestodes of *Echinococcus granulosus *or *E. multilocularis*, and eggs released from *Schistosoma mansoni *and *S. japonicum *localise primarily in the liver where it is thought that host immune responses direct fibrogenesis. The resultant fibrosis of the liver may be associated with severe host pathology in schistosomiasis [1], or in echinococcosis with a peri-parasitic extracellular matrix (ECM)-rich 'barrier' that acts to restrict cyst growth but, paradoxically, may prevent successful drug interactions with the parasite [2].

**Figure 1:**
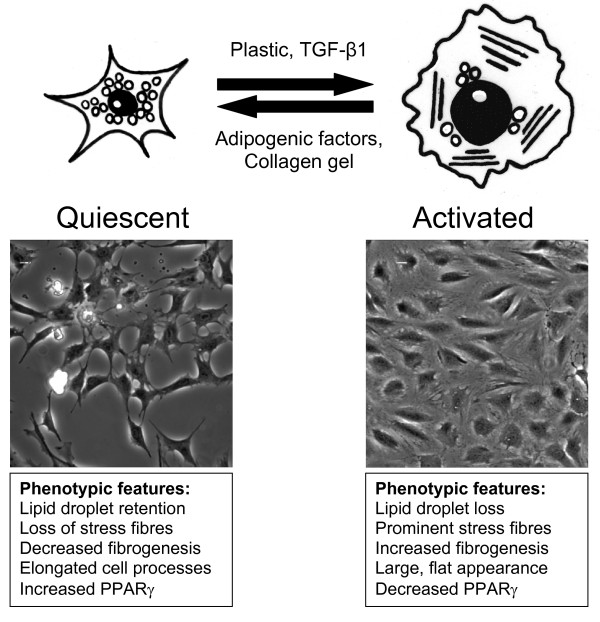
**Summary of the process of hepatic stellate cell (HSC) trans-differentiation**. The imaged cells are LX-2 cells taken after 72hrs growth with adipogenic factor induced quiescence on the left and activated cells on the right. The quiescent HSC phenotypically has a small cell body with cellular processes extending into the growth area around the cell. Lipid droplets are observed in the cells that are characterised by a lack of αSMA stress fibres, low expression of fibrosis associated genes (Col1A1, CTGF and αSMA) and increased expression of PPAR-γ. Upon activation, the cells lose their ability to store lipid droplets and have a large flat appearance. αSMA stress fibres are observed in the cells and gene expression of fibrosis associated genes are increased while PPAR-γ expression is reduced.

In diseases characterised by fibrosis there is excessive scarring which is caused by production, deposition and cellular contraction of ECM [3]. This occurs when the normal wound healing response, which after injury is initiated to synthesise new connective tissue, fails to terminate [3]. This process results in proliferation and migration of mesenchymal fibroblasts to the area of injury where they synthesise elevated levels of matrix proteins such as collagen and fibronectin [3]. In liver fibrosis the mesenchymal cells are the hepatic stellate cells (HSC) (formally called Ito cells) which store vitamin A located in the space of Disse within the liver sinusoid [4]. For the liver to function normally, solutes and growth factors in the blood plasma can enter this space by passing through fenestrae in liver endothelial cells (LSECs) where they come into contact with hepatocytes. Upon activation, HSCs undergo a process of trans-differentiation from a vitamin A-storing cell to a myofibroblast phenotype [5]. A scar-like matrix is produced by the myofibroblasts that impedes the flow of solutes to the hepatocytes and, as a result of accumulating ECM products, the LSECS undergo defenestration and the hepatocytes lose there microvilli thereby stopping the liver functioning effectively [4]. The process of trans-differentiation of the HSC is summarised in Figure 1.

The process of fibrogensis in the liver has been well defined over recent years with HSC activation central to this. The first phrase of fibrosis is initiation in which insult to the liver results in activation of the HSCs into myofibroblasts [5]. There are many known types of liver insult ranging from viral infection (due to hepatitis B, C and D), autoimmunity (primary biliary cirrhosis, autoimmune hepatitis), inherited diseases (cystic fibrosis, hereditary haemachromotosis), dietary (non-alcoholic fatty liver disease) or chemical (alcohol) causes, and parasitic infection, the details of which are beyond the scope of this article but have been summarised by Wallace *et al *[6]. After the first initiation of fibrogenesis whereby the HSCs proliferate, become contractable, produce fibrogenic ECM components, alter matrix degradation, recruit new myofibroblasts and become involved in inflammatory signalling [5]. All these responses are tightly controlled by cytokine production both from the HSCs themselves as well as neighbouring cells.

Myofibroblasts are regarded by many as the cell with a central role in liver fibrogenesis and as the main cellular source of ECM components in liver fibrogenesis [7]. There is still some argument regarding the precise origin of these cells but many agree that during fibrosis they can originate from different sources. They can derive from quiescent HSCs which are located within the centrilobular and perisinusoidal regions of the liver lobule but also from portal track fibroblasts within the periportal regions of the lobule [6]. These sub-populations of myofibroblasts have been demonstrated by differential staining of myofibroblast markers [8]. Myofibroblasts have been demonstrated to be additionally derived from epithelial-mesenchymal transition [9] and from circulating fibroblasts called fibrocytes [10].

It is widely accepted that kupffer cells have a role in liver fibrogenesis. In liver fibrosis the number of macrophages increase and they are found to be located around areas of tissue damage and fibrosis [6]. Alternately activated macrophages produce proline and arginase-1 which contribute to collagen synthesis [11]. Additional evidence supporting the role of kupffer cells in fibrogensis has been demonstrated in studies using the *in vivo *CCL4 model of fibrosis in mice which showed that inhibiting kupffer cell function with gadolinium chloride [12] resulted in reduced fibrosis.

Granuloma formation in schistosomiasis has been described as a two stage process with a pre-granulomatous stage, characterised by disorganised aggregation of cells, and a granulomatous stage in which they become organised [13]. The process is tightly controlled by the host immune response. Early in the acute phase of infection, the Th1 response is predominant in patients and is characterised by high levels of INF-γ and TNF-α, and is associated with large granulomas around the eggs in the liver [14]. The release of eggs gradually causes a switch to a Th2 response that is characterised by high levels of IL-4, IL-13 and IL-10 [14]. IL-4 and IL-13 have been demonstrated to be important in the induction of the Th2 response in schistosomiasis [11]. It is believed that this switch in response is important in terms of disease severity as IL-4-/- knock-out mice die due to sepsis as haemorrhagic lesions in the gut mucosa increase exposure of the host to bacteria and bacterial toxins from the gut [11,15]. It has been demonstrated that the eggs themselves can stimulate the Th2 response with an IL-4 inducing factor (IPSE) being produced by the egg that stimulates activation of basophills and the expression of IL-4 and IL-13 [16]. Omega-1 has also been identified as a major component found in soluble egg antigen (SEA) that can condition dendritic cells for Th2 polarisation [17].

In the chronic form of schistosomiasis, the immune response is generally down-regulated but failure to do so in some individuals leads to development of hepatosplenic disease [11]. Different models of down-regulation have been suggested. B-cells have been implicated in this process [11]. Mice deficient in B cells fail to undergo Th2 down-regulation and, as a result, develop a more severe pathology with failure to decrease granuloma size during chronic infection [18]. Another role in down-regulation has been demonstrated by the IL-13 receptor α2 (IL-13r α2) which has been observed to down-modulate granulomatous inflammation and prolong host survival in mice [19]. Its expression is higher both in mice and human patients with schistosomiasis suggesting it does have an important role in disease progression [19].

Fibrosis is the most serious pathology associated with schistosomiasis but little is known of its initiation. The type of fibrosis observed in schistosomiasis is periportal fibrosis, also known as Symmer's pipe-stem fibrosis, named after W. Symmers who first described the fibrosis in the liver of a patient chronically infected with *S. mansoni *[20]. Four stages have been suggested in the process of fibrosis in schistosomiasis. These are: recruitment of fibroblasts and/or differentiation of HSC; proliferation of the HSC; secretion of ECM molecules; and ECM remodelling [21]. In chronic schistosomiasis, the rate of collagen accumulation decreases and a change in the type of collagen accumulated can occur from type 1 to type 3 as demonstrated in mice infected with *S. japonicum *[22] . In progressive fibrosis the main type of collagen produced is type 1 [23].

The development of fibrosis appears to depend on the interaction of many different factors although an immune response polarised towards a Th2 response is recognised as being associated with schistosome-induced fibrogenesis [11,15,24,25]. Indeed, high levels of IL-5 and IL-13 have been observed in patients with more serious fibrosis [24]. IL-13 has, additionally, been demonstrated to be pro-fibrogenic in mice [15] and to be associated with stimulation of collagens 1, 3 and 5 [11]. IL-13 has been shown to promote collagen production in a human HSC cell line [26]. In contrast, cytokines (TNF-α, IL-12, INF-γ) and NO, associated with the Th1 response, have been observed to inhibit fibrosis in schistosomiasis [11].

Different populations of myofibroblasts have been observed to be involved in the development of fibrosis in the granuloma with inward collagenic growth being associated with myofibroblasts from the portal track while outward growth is attributed to myofibroblasts originating from HSC differentiation [13]. Macrophages have been demonstrated to have an important role in the development of fibrosis in schistosomiasis. The Th1 cytokines, INF-γ and TNF-α can classically activate macrophages to produce NOS-2 which in mice is associated with smaller granulomas and less fibrosis [11]. In contrast, macrophages stimulated by Th2 cytokines, especially IL-13, become alternatively activated and produce arginase-1 (arg-1) and proline, higher levels of which are associated with larger granulomas and increased fibrosis [11]. Other factors associated with the development of fibrosis in schistosomiasis are repeated exposure [25] and intensity of infection [27]. In a study on human patients, an increase in egg deposition in chronically infected individuals was observed to shift the immune response from a Th0 to a Th2 response and this correlated with increased levels of IL-10 [27]. Another study on *S. mansoni-*infected baboons demonstrated that repeated infection and treatment primed lymphocytes to produce increased levels of TGF-β and IL-4 which are known pro-fibrogenic cytokines [25].

## Evidence for a role for HSC in parasitic disease

Little is known about the role of the HSC in schistosome-induced fibrosis, although it is thought that this cell is one of the main sources of collagen deposition and it plays a role in extracellular matrix remodelling in schistosomiasis [28]. HSC have been identified in the periphery of egg granulomas in murine and human *S. japonicum *infection where myofibroblasts were shown to be present in fibrotic patients with end-stage disease [29]. In the mouse study, the presence of HSC was shown using immunohistochemical techniques. Staining for markers such as desmin and αSMA occurred at the edges of granulomas with HSC activation detectable after 6 weeks post-*S. japonicum *infection, coinciding with the TH2 shift and down-modulation of granuloma size [29]. Peak activation of HSC was observed in mice between 8-10 weeks post-infection, measured by αSMA expression [29]. A further study of the *S. japonicum *model again demonstrated staining for αSMA positive cells in the fibrotic area of granulomas [30]. Staining in this model coincided with the up-regulation of genes associated with hepatic fibrosis and HSC activation as well as chemokines associated with HSC recruitment [30]. In a separate study the presence of activated HSC in human *S. mansoni *infection was demonstrated [31].

Increased levels of somatostatin, which can inhibit HSC activation, in patients infected with *S.mansoni *has been shown to be associated with reduced fibrosis [32]. In rabbits infected with *S. japonicum*, HSC activation was found to be characteristic of the resulting fibrosis and could be inhibited by administration of prostaglandin E1 which inhibits HSC activation [33]. Leptin, and its receptor, is found to be expressed in granulomas of *S.mansoni-*infected mice [13]. Leptin is expressed in activated HSC and increases the cells ability to produce collagen type 1 by enhancing the effect of the TGF-β type 2 receptor [34]. It is noteworthy that leptin deficiency decreases fibrosis in *S.mansoni *infection in mice further indicating a role for HSC in the development of fibrosis [35]. Additional evidence for the involvement of the HSC is supported in a murine study in which the Chinese traditional medicine, heluoshugan, was observed to decrease collagen production by inhibiting their activation [36]. Paeoniflorin, which has anti-inflammatory, anti-allergic, and immunoregulatory effects and is commonly used in Chinese herbal prescriptions to treat hepatic disorders, has been demonstrated by Li et al. [37] to reduce fibrosis in murine *S. japonicum *infection as well as inhibit collagen synthesis in IL-13-stimulated HSC. Previously this group had demonstrated that mouse peritoneal macrophages stimulated by SEA from *S. japonicum *produced TGF-β that was able to stimulate collagen production in HSC [37]

Vuitton's group [38,39] postulated a possible role for HSC in alveolar echinococcosis. Using various imaging techniques they demonstrated that myofibroblasts were the main cell type in areas of developing liver fibrosis; in some regions of the liver the parenchyma was replaced with parasitic lesions, acellular necrosis and fibrosis and that in these areas HSC were the only cellular remnants present. Additionally, TGF-β is secreted into the granuloma area around *E. multilocularis *[40] which is a well studied model of HSC activation. Activated HSC have, additionally, been identified in *Mesocestoides vogae *cestode infection in mice with these cells forming a cellular layer in proximity to the larval surface and that in progressive infection the population of SMA-positive myofibroblasts increased in fibrous lesions around the larvae [41].

## The HSC as a fibrogenesis model

The HSC has been widely used as an *in vitro *model of fibrogenesis [6]. This has allowed the process of trans-differentiation to be studied in this cell as under normal cell culture conditions quiescent HSC spontaneously activate into the myofibroblast phenotype. Primary rat HSC have been used extensively as an *in vitro *model and can be obtained from rat liver tissue by perfusion with collagenase and pronase through the portal vein in order to obtain a suspension of non-parenchymal liver cells [42]. Then, due to a high fat content, HSCs can be purified using their low buoyant density; the yield of HSCs is dependent upon rat age, body weight and nutritional status, with older animals fed on a normal diet or younger rats fed with a vitamin A-supplemented diet providing the highest number of cells [42]. Whereas much valuable information has been obtained using rat HSCs, it is important to undertake similar studies with human HSCs in order to validate whether the mechanisms established in rodent models have relevance for human biology [43]. Some work has been carried out using modified rat cell isolation procedures to isolate primary human HSCs from liver wedge sections obtained during liver surgery [42]. Obtaining human HSCs is difficult, however, as wedge sections do not allow adequate perfusion of the tissue which results in low yields of HSC [42], and the availability of human tissue suitable for isolation is infrequent and unpredictable [43]. Additionally, some groups report that they find human HSC isolated cells senesce between passages 2-5 [6]. Indeed, Wallace et al. [6] noted that in their studies only 2 cultures remained proliferative for over 5 passages, and they suggested this may be due to the fact the cells were isolated mainly from elderly patients.

This relative lack of success has led to the development of human HSC cell lines. The LX-2 cell line is a recently developed human immortalised cell line, developed alongside a LX-1 cell line, which has been shown to retain key features of HSC biology [43]. These cells were developed by transfecting primary cells with the pRSVTag plasmid which encodes the SV40 large T antigen controlled by the rous sarcoma virus [43]. LX-1 cells were then established from the primary T antigen culture while the LX-2 cells were established from a sub-line of the LX-1 cells that were able to grow in reduced serum conditions [43]. Another cell line that has been developed is the TWNT-4 cell line immortalised by retrovirally introducing human telomerase reverse transcriptase (hTERT) into LI90 cells established from a human liver mesenchymal tumour [42]. These cells are considered an activated HSC type with cells expressing platelet derived growth factor receptor-β (PDGF-Rβ), αSMA and type 1 collagen [44]. Another cell line was developed by infecting primary HSC with a retrovirus expressing hTERT [45]; the cells were demonstrated to retain the features of primary HSC [45].

TGF-β has been used in numerous studies with HSC and has been shown to promote differentiation into the myofibroblast phenotype [3,46] and also within the LX-2 cell line [43]. The substrate that HSC are cultured on has, additionally, been observed to regulate their phenotype. Primary rat HSC have been shown to have a phenotype reverted back to a more quiescent form by their growth on or within type 1 collagen gels compared to cells grown on cell culture plastic [47]. Culture on collagen gels results in cells that extend their cellular processes into the growth area containing vitamin A, with the cells overall forming a mesh-like structure [48]. Matrigel has been demonstrated to cause quiescence in rat HSC and restore their vitamin A storing ability, while culture on cell culture plastic results in activation of the cells over time [49].

A major role of the HSC is in the storage of vitamin A as fat droplets [42]. This has highlighted similarities with adipocytes, the phenotype of which can be activated into a fibroblast-type phenotype by cytokines such as PDGF, TGFα and Leptin [50]. Reversal back into an adipocyte has been achieved by treating the activated adipocytes with a mixture of adipogenic factors [50]. The reversal back into fat-storing type cells has been associated with an increase in expression of PPARγ, of which a reduced level is observed in activated HSC [50]. This treatment has been used on activated primary rat HSC and has been shown to cause a decrease in collagen synthesis, an increase in PPARγ expression and restoration of the adipogenic phenotype of quiescent HSC [50,51]. Adipogenic factors have additionally, been used on LX-2 cells and were observed to cause an increase in PPARγ expression and cause accumulation of fat droplets demonstrated by Oil-Red O staining [52]. It was further demonstrated that use of a PPARγ agonist, SB431542, inhibits TGF-β activation of the cells [52].

## Can parasites interact with HSC directly

Until recently, there were no studies that had investigated the direct effects of schistosome egg antigens on the trans-differentiation status of HSC. Anthony et al. [53] used the LX-2 line as a model for co-culture experiments with viable *S. mansoni *eggs. Not only were the eggs able to directly affect the trans-differentiation process of the cells but, surprisingly, they caused regression of the LX-2 cell line into quiescent vitamin A-storing cells characterised by increased expression of PPAR-γ. This finding may help clarify the progression of fibrosis in schistosomiasis as this was the first report of a direct interaction between a parasite and HSC. It is noteworthy that the anti-diabetic thiazolidinedione drug rosiglitazone reduces hepatic fibrosis in murine schistosomiasis and works by activating the PPAR-γ ligand [54]. In the studies identifying the HSC as key in granuloma formation [29,31], it was noted that these cells where observed at the periphery of the granuloma and not in the immediate vicinity of the eggs. Additionally, it is not until the egg has been destroyed that fibrosis is observed throughout the granuloma area. It may be that the eggs themselves cause this down-regulation of activation status and HSC can only be activated at the periphery of the granuloma from a descending concentration gradient of egg antigen or in the absence of eggs from the granuloma after their destruction. It may be that other parasitic diseases such as echinococcosis, in which liver fibrosis is a factor, may be able to interact with HSC where the ability to inhibit fibrogenesis may favour establishment of the parasite in the mammalian host.

## Conclusions and the future

There is growing evidence that the HSC may be critically important in the progression of parasite-induced diseases, and that the interaction of parasites or parasite antigens with this cell can provide new insights in our understanding of the pathogenesis of schistosomiasis and alveolar echinoccocosis. To investigate this further, both *in vivo *and *in vitro *approaches are possible. For example, immunohistochemisty could be used to look at the presence of HSC in experimental infections which could then be correlated with the host immune response. This approach would allow identification of the response(s) associated with HSC activation and severe pathology that may reveal mechanisms for designing possible immunotherapeutic strategies including pathology-limiting vaccines. The HSC provides an unique *in vitro *model of liver fibrogenesis that has been extensively utilised in studies of many liver diseases [55-57]. Use of the cell in an *in vitro *setting has been made easier with the availability of new cells lines of human origin. However, this model has generally been overlooked in relation to the study of parasite-induced fibrogenesis. Investigating the response of the cells to parasite antigens using techniques such as microarrays, RNAi and flow cytometry could reveal new insights into the process of parasite-induced fibrogenesis.

## List of abbreviations

ECM: Extracellular Matrix; HSC: Hepatic Stellate Cells; LSECs: Liver Endothelial Cells; CCL_4_: Carbon Tetrachloride; INF-γ: Interferon-gamma; TNF-α: Tumour Necrosis Factor-alpha; IPSE: IL-4 Inducing Factor; SEA: Soluble Egg Antigen; IL-13r α2: IL-13 receptor α2; NO: Nitric Oxide; NOS-2: Nitric Oxide Synthase; arg-1: Arginase-1; TGF-β: Transforming Growth Factor-beta; αSMA: alpha Smooth Muscle Actin; hTERT: Human telomerase reverse transcriptase; PDGF: Platelet Derived Growth Factor; PPAR-γ: Peroxisome Proliferator-Activated Receptor gamma.

## Competing interests

The authors declare that they have no competing interests.

## Authors' contributions

BA drafted the manuscript and was the major contributor to the review article. JTA contributed to the article draft. YSL jointly conceived of the study and its design. DPM conceived of the study and its design, and contributed to the manuscript draft.
